# Epistasis Analysis for Estrogen Metabolic and Signaling Pathway Genes on Young Ischemic Stroke Patients

**DOI:** 10.1371/journal.pone.0047773

**Published:** 2012-10-24

**Authors:** Yi-Chen Hsieh, Jiann-Shing Jeng, Huey-Juan Lin, Chaur-Jong Hu, Chia-Chen Yu, Li-Ming Lien, Giia-Sheun Peng, Chin-I Chen, Sung-Chun Tang, Nai-Fang Chi, Hung-Pin Tseng, Chang-Ming Chern, Fang-I Hsieh, Chyi-Huey Bai, Yi-Rhu Chen, Hung-Yi Chiou, Formosa Stroke Genetic Consortium (FSGC), Jiann-Shing Jeng, Sung-Chun Tang, Shin-Joe Yeh, Li-Kai Tsai, Shin Kong, Li-Ming Lien, Hou-Chang Chiu, Wei-Hung Chen, Chyi-Huey Bai, Tzu-Hsuan Huang, Lau Chi-Ieong, Ya-Ying Wu, Rey-Yue Yuan, Chaur-Jong Hu, Jau- Jiuan Sheu, Jia-Ming Yu, Chun-Sum Ho, Chin-I Chen, Jia-Ying Sung, Hsing-Yu Weng, Yu-Hsuan Han, Chun-Ping Huang, Wen-Ting Chung, Der-Shin Ke, Huey-Juan Lin, Chia-Yu Chang, Poh-Shiow Yeh, Kao-Chang Lin, Tain-Junn Cheng, Chih-Ho Chou, Chun-Ming Yang, Giia-Sheun Peng, Jiann-Chyun Lin, Yaw-Don Hsu, Jong-Chyou Denq, Jiunn-Tay Lee, Chang-Hung Hsu, Chun-Chieh Lin, Che-Hung Yen, Chun-An Cheng, Yueh-Feng Sung, Yuan-Liang Chen, Ming-Tung Lien, Chung-Hsing Chou, Chia-Chen Liu, Fu-Chi Yang, Yi-Chung Wu, An-Chen Tso, Yu- Hua Lai, Chun-I Chiang, Chia-Kuang Tsai, Meng-Ta Liu, Ying-Che Lin, Yu-Chuan Hsu, Chih-Hung Chen, Pi-Shan Sung, Chang-Ming Chern, Han-Hwa Hu, Wen-Jang Wong, Yun-On Luk, Li-Chi Hsu, Chih-Ping Chung, Hung-Pin Tseng, Chin-Hsiung Liu, Chun-Liang Lin, Hung-Chih Lin, Chaur-Jong Hu

**Affiliations:** Principal Investigator; Principal Investigator; Principal Investigator; Principal Investigator; Principal Investigator; Principal Investigator; Principal Investigator; Principal Investigator; (Principal Investigator; Principal Investigator; 1 School of Public Health, Taipei Medical University, Taipei, Taiwan; 2 Stroke Center and Department of Neurology, National Taiwan University Hospital, Taipei, Taiwan; 3 Department of Neurology, Chi-Mei Medical Center, Tainan, Taiwan; 4 Department of Neurology, Taipei Medical University Hospital and Shuang Ho Hospital, Taipei, Taiwan; 5 Department of Neurology, Shin Kong Wu Ho-Su Memorial Hospital, Taipei, Taiwan; 6 Department of Neurology, Tri-Service General Hospital, Taipei, Taiwan; 7 Department of Neurology, Wanfang Hospital, Taipei Medical University, Taipei, Taiwan; 8 Department of Neurology, Lotung Poh-Ai Hospital, I-Lan, Taiwan; 9 Department of Neurology, Taipei Veterans General Hospital, Taipei, Taiwan; 10 Department of Medicine, School of Medicine, National Yang-Ming University, Taipei, Taiwan; 11 Dr. Chi-Hsing Huang Stroke Research Center, Taipei Medical University, Taipei, Taiwan; University of Queensland, Australia

## Abstract

**Background:**

Endogenous estrogens play an important role in the overall cardiocirculatory system. However, there are no studies exploring the hormone metabolism and signaling pathway genes together on ischemic stroke, including sulfotransferase family 1E (SULT1E1), catechol-*O*-methyl-transferase (COMT), and estrogen receptor α (ESR1).

**Methods:**

A case-control study was conducted on 305 young ischemic stroke subjects aged ≦ 50 years and 309 age-matched healthy controls. SULT1E1 -64G/A, COMT Val158Met, ESR1 *c.*454−397 T/C and *c.*454−351 A/G genes were genotyped and compared between cases and controls to identify single nucleotide polymorphisms associated with ischemic stroke susceptibility. Gene-gene interaction effects were analyzed using entropy-based multifactor dimensionality reduction (MDR), classification and regression tree (CART), and traditional multiple regression models.

**Results:**

COMT Val158Met polymorphism showed a significant association with susceptibility of young ischemic stroke among females. There was a two-way interaction between SULT1E1 -64G/A and COMT Val158Met in both MDR and CART analysis. The logistic regression model also showed there was a significant interaction effect between SULT1E1 -64G/A and COMT Val158Met on ischemic stroke of the young (P for interaction = 0.0171). We further found that lower estradiol level could increase the risk of young ischemic stroke for those who carry either SULT1E1 or COMT risk genotypes, showing a significant interaction effect (P for interaction = 0.0174).

**Conclusions:**

Our findings support that a significant epistasis effect exists among estrogen metabolic and signaling pathway genes and gene-environment interactions on young ischemic stroke subjects.

## Introduction

Previous population-based epidemiological studies reported stroke incidence rate to be lower in women during midlife than that in either older aged women or men [1], [2]. The relatively high risk of ischemic stroke in premature menopause or early menopause women has drawn attention to the role of estrogen in cardiovascular disease. In addition to experimental studies demonstrating the protective roles of estrogens in many forms of cardiovascular and cerebrovascular diseases [3]–[5], a large volume of epidemiological and observational findings also indicate that exposure to endogenous estrogen has been postulated to be protective for stroke in premenopausal women [6], [7]. It is well established that the beneficial effects of estrogen on vascular system includes enhancing nitric oxide (NO) production and vascular relaxation [8], [9], accelerating endothelial growth factor after vascular injury [4], [10], improving serum lipid concentration [11]–[13]. Since abundant evidence demonstrates that estrogen might play an important role in the overall cardiocirculatory system, understanding the estrogen metabolic and signaling pathway in relation to vascular disease may shed light on the role of estrogen in ischemic stroke pathogenesis. In this study, we focused on 3 genes involved in steroid hormone metabolism and signaling: sulfotransferase family 1E (SULT1E1), catechol-*O*-methyl-transferase (COMT), and estrogen receptor α (ESR1).

Sulfotransferase enzyme encoded by SULT1E1 (OMIM600043) catalyzes the sulfate conjugation of estrone (E1), 17β-estradiol (E2), catecholestrogens and 2-methoxyestradiol [14]–[16], which is a major pathway for estrogen metabolism in humans [15], [17]. The human SULT1E1 gene is approximately 20 kb in length, consists of eight exons, and maps to chromosome 4q13 [18]. There are 23 polymorphisms identified in the SULT1E1 gene, and most of them are in low allele frequencies except -64G/A in exon1 and other three in intron [19]. Recent researches further indicated that SULT1E1 -64G/A (rs3736599) in the promoter region is positively correlated with endometrial cancer risk [20], [21]. COMT, encoding catechol-*O*-methyltransferase, is a phase II enzyme that catalyzes the inactivation of the major metabolites of estrogen [22]. A functional single nucleotide polymorphism (SNP) for COMT gene (OMIM16790), mapping to chromosome 22q11 in exon 4 (Val158Met, rs4680), has been identified the Met (A) allele is linked to a variant of the COMT gene, which results in 3- to 4-fold decreased enzyme activity of COMT [23]. Several evidences demonstrated that COMT Val158Met is significantly associated with breast cancer [24], [25]. It is biologically reasonable to hypothesize that women who carry the mutant COMT Met allele may have higher risks of ischemic stroke. In addition to metabolism of estrogen, estrogens exert their effects by binding to specific estrogen receptors α (encoded by ESR1) and β (encoded by ESR2). The human ESR1 gene (OMIM133430) is located on chromosome 6q25, comprising of 8 exons and 7 introns. Considerable studies focusing on ESR1 *c.*454−397 T/C (rs2234693) and *c.*454−351 A/G (rs9340799) polymorphisms in intron 1 are most widely discussed, and the studies found that these two SNPs are associated with ischemic stroke [26], [27], cardiovascular disease [28], [29], and atherosclerosis [30].

Since role of estrogen in cerebrovascular pathophysiology and ischemia is an important area of ongoing investigation, to our knowledge, there is no study focusing on both the estrogen metabolism and signaling pathway genes. The present study was carried out with the aim to determine whether SULT1E1 -64G/A, COMT Val158Met, ESR1 *c.*454−397 T/C and *c.*454−351 A/G genes are associated with ischemic stroke of the young and to further explore the gene-gene and gene-environment interactions for young ischemic stroke patients.

## Methods

The study was approved by the institutional review board of Taipei Medical University and the participated hospitals, including National Taiwan University Hospital, Shuang Ho Hospital, Chi-Mei Medical Center, Shin Kong Wu Ho-Su Memorial Hospital, Tri-Service General Hospital, Wanfang Hospital, Lotung Poh-Ai Hospital, and Taipei Veterans General Hospital. Written, informed consent was obtained from all participants and/or their relatives.

**Table 1:** Demographic and clinical characteristics of ischemic stroke and healthy controls.

Characteristic	Stroke patients (n = 305)		healthy Controls (n = 309)		P-value
Age, mean (SD), y	43.7	6.0	43.6	5.6	0.7626
Male, n (%)	217	71.2	221	71.5	0.9896
Hypertension, n (%)	179	59.7	74	24.0	<0.0001
Diabetes mellitus, n (%)	88	29.1	20	6.5	<0.0001
Ever smoking, n (%)	161	54.0	120	39.1	0.0003
Ever drinking, n (%)	92	30.8	47	15.2	<0.0001
Body mass index, mean (SD), kg/m^2^	25.5	4.6	24.3	3.0	0.0003
Waist-hip ratio, mean (SD)	0.91	0.07	0.84	0.08	<0.0001
Fasting glucose, means (SD), mmol/L	130.1	75.3	98.9	23.1	<0.0001
HDL-C, mean (SD), mmol/L	1.09	0.42	1.24	0.33	<0.0001
LDL-C, mean (SD), mmol/L	3.35	1.16	3.08	0.73	0.0034
Triglyceride, mean (SD), mmol/L	1.90	1.46	1.57	1.16	0.0026
Cholesterol, mean (SD), mmol/L	5.04	1.47	5.04	0.90	0.9318
Estradiol level, mean (SD)*	1.4	0.3	1.5	0.4	0.1728
TOAST, n (%)					
Large artery atherosclerosis	77	25.2			
Small vessel occlusion	99	32.5			
Cardioembolism	16	5.2			
Specific pathogenesis	33	10.8			
Undetermined pathogenesis	42	13.8			
Missing	38	12.5			

* Estradiol level was shown in log transformed value.

### Study Subjects

In this study, there were 305 ischemic stroke subjects aged ≦ 50 years recruited from 2005 to 2010, including 217 males and 88 females. Details of the participants’ enrollment were described elsewhere [31]. In brief, this case-control study was conducted by the Formosa Stroke Genetic Consortium (FSGC) in Taiwan. FSGC is a platform for hospital collaborations on studies related to the molecular biology of cerebrovascular diseases. A standard operation manual of FSGC was established by an expert panel including 5 stroke neurologists and 3 epidemiologists after a series of consensus conferences. All staffs from the participating hospitals were trained on the standard procedure of case enrollment, including structured questionnaire and blood sample collection. All collaborating hospitals participated in the FSGC since 2005. The diagnostic criteria of stroke have been described in previous study [32]. The definition of ischemic stroke is an onset of focal neurological deficit with signs or symptoms persisting longer than 24 hours with or without acute ischemic lesion(s) on brain CT, or with acute ischemic diffusion-weighted imaging lesion(s) on MRI that corresponded to the clinical presentations. TIA is defined as a transient focal neurologic deficit of ischemic causes that resolves within 24 hours. The subtypes of ischemic stroke were categorized according to the Trial of Org 10172 in Acute Stroke Treatment (TOAST) criteria [33]. There were 2736 healthy subjects recruited as possible controls, including 1637 individuals from a community-based prospective study of the nutrition health education program in Taipei City [34] and 1099 subjects who underwent physical examinations at TMUH during 2008–2009. All participants were recruited if they agreed to write informed consent. Among them, 53 subjects with prevalent stroke were excluded. 309 subjects were randomly selected as age-matched controls in the remaining 2683 candidates. The distribution of age, gender, education levels were similar between the selected subjects and the remaining candidates.

**Table 2:** Genotype and allelic frequencies of the SULT1E1, COMT, and ESR1 genes in ischemic stroke patients and controls and the estimated odds ratio and 95% confidence interval of ischemic stroke risk.

		Total					Female					Male				
		Cases/Controls	OR (95% CI)	P-value	P for trend	P for permutation	Cases/Controls	OR (95% CI)	P-value	P for trend	P for permutation	Cases/Controls	OR (95% CI)	P-value	P for trend	P for permutation
SULT1E1	GG	142/150	1.0		0.1264	0.4165	42/47	1.0		0.2258	0.6560	100/103	1.0		0.3094	0.7692
-64G/A	GA	123/141	0.90(0.60–1.36)	0.6315			32/33	1.37(0.58–3.26)	0.4771			91/108	0.77(0.48–1.24)	0.2773		
rs3736599	AA	36/17	3.39(1.60–7.17)	0.0014			13/7	3.52(0.91–13.60)	0.0679			23/10	3.50(1.35–9.05)	0.0099		
	G allele	407/441	1.0				116/127	1.0				291/314	1.0			
	A allele	195/175	1.35(1.00–1.84)	0.0521			58/47	1.67(0.91–9.08)	0.0986			137/128	1.21(0.85–1.73)	0.2970		
COMT	GG	152/179	1.0		0.0231	0.0919	38/56	1.0		0.0033	**0.0120**	114/123	1.0		0.3818	0.8459
Val158Met	GA	119/111	1.11(0.74–1.67)	0.6235			40/28	1.71(0.71–4.15)	0.2356			79/83	0.94(0.59–1.52)	0.8079		
rs4680	AA	30/18	1.87(0.86–4.05)	0.1149			9/3	4.38(0.78–24.65)	0.0937			21/15	1.59(0.66–3.80)	0.3004		
	G allele	423/469	1.0				116/140	1.0				307/329	1.0			
	A allele	179/147	1.21(0.88–1.67)	0.2362			58/34	1.93(1.00–3.76)	0.0517			121/113	1.05(0.73–1.52)	0.7953		
ESR1	TT	114/114	1.0		0.7036	0.9901	31/32	1.0		0.7363	0.9946	83/82	1.0		0.8157	0.9992
c.454 -397T/C	TC	142/157	1.00(0.66–1.52)	0.9895			43/44	2.33(0.87–6.24)	0.0913			99/113	0.87(0.53–1.41)	0.5622		
rs2234693	CC	45/37	1.72(0.93–3.19)	0.0868			13/11	2.37(0.66–8.59)	0.1878			32/26	1.63(0.77–3.39)	0.1958		
	T allele	370/385	1.0				105/108	1.0				265/277	1.0			
	C allele	232/231	1.25(0.94–1.58)	0.1297			69/66	1.54(0.84–2.80)	0.1598			163/165	1.19(0.84–1.68)	0.3209		
ESR1	AA	176/188	1.0		0.8241	0.9992	45/56	1.0		0.2585	0.6890	131/132	1.0		0.3624	0.8143
c.454 -351A/G	AG	113/98	1.18(0.78–1.78)	0.4334			40/28	1.89(0.82–4.38)	0.1365			73/70	1.01(0.62–1.64)	0.9641		
rs9340799	GG	12/23	0.88(0.37–2.10)	0.7655			2/4	1.08(0.13–8.69)	0.9460			10/19	0.80(0.31–2.11)	0.6569		
	A allele	465/474	1.0				130/140	1.0				335/334	1.0			
	G allele	137/144	1.10(0.79–1.54)	0.5588			44/36	1.42(0.73–2.76)	0.3025			93/108	1.00(0.68–1.47)	0.9930		

OR was adjustment for age, education level, hypertension, diabetes mellitus, dyslipidemia, obesity, and cigarette smoking.

### Data Collection and Assessments

All participants were informed to draw venous blood for biochemical test, including cholesterol (CHOL), triglyceride (TG), high-density lipoprotein cholesterol (HDL-C), fasting glucose, and estradiol level. Blood samples were collected when patients were confirmed as ischemic stroke and when controls agreed to participate in the study. Fasting serum CHOL, TG, HDL-C, and glucose concentrations were measured by an automatic analyzer (UniCel DXC 800, BeckMan). Low-density lipoprotein cholesterol (LDLC) was calculated using Friedewald formula [35]. Laboratory assay for estradiol was measured by radio immunoassay (RIA). The lower limit of quantitation of estradiol level was 2 pg/ml. Duplicate samples were included for 10% of the subjects for quality control purposes. Samples were labeled in such a way that laboratory personnel were unaware of the case-control status of the samples and the identity of the duplicates. Log-transformed was done for serum estradiol level to follow a normal distribution before executing association analyses. In this study, high or low serum estradiol level was defined based on the median level of healthy controls which were 1.68 and 1.38 for female and male, respectively. The anthropometrical measurements were assessed by trained technicians. Body mass index (BMI) was defined as the individual’s body weight (kg) divided by square of their height (m^2^). Waist-to-hip ratio (WHR) was computed using index of waist circumference divided by the hip circumference. Obesity was defined as waist circumstances ≧80 cm for female and ≧90 cm for male or BMI>27 kg/m^2^.

**Figure 1:**
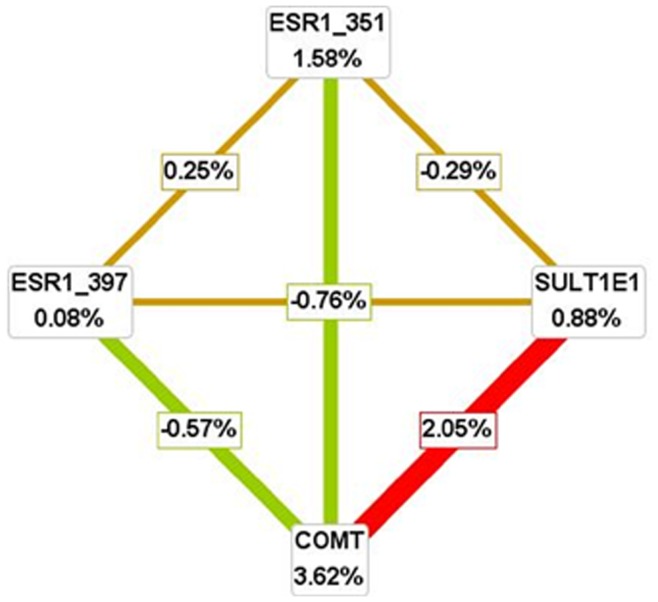
Interaction map for young ischemic stroke risk among females. Values in nodes represent information gain (IG) of individual attribute (main effect). Values between nodes are IGs of each pairwise combination of attributes (interaction effects). A positive entropy (plotted in red or orange) indicates interaction while a negative (plotted in green) indicates redundancy.

### DNA Collection and Genotyping

Genomic DNA was extracted from EDTA-anticoagulated peripheral blood leukocytes by the phenol/chloroform method and then stored at −80°C until use. Genotyping was carried out by polymerase chain reaction (PCR) and restriction fragment length polymorphism (RFLP). Genotyping assays were performed using standard protocol in a total volume of 40 ul and 1 to 2 ng of sample DNA was used per assay. Genotyping was performed by laboratory technicians blinded to the case-control status. As a quality control, we repeated to validate the genotyping on 10% of the samples and the concordance rate for replicate samples was 100%. The overall genotyping success rates were >98%.

**Table 3:** Summary of MDR gene-gene interaction results.

Model	Training Bal. Acc. (%)	Testing Bal. Acc. (%)	Cross-validationConsistency	p-value*
COMT	0.5396	0.5769	7/10	0.0140
SULT1E1, COMT	0.5720	0.5722	9/10	0.0240
COMT, SULT1E1, ESR1 (c.454 -397T/C)	0.5989	0.5951	7/10	0.0020
SULT1E1, COMT, ESR1 (c.454 -351A/G), ESR1 (c.454 -397T/C)	0.6279	0.5823	10/10	0.0080
**Female**				
COMT	0.6034	0.6264	10/10	0.0370
SULT1E1, COMT	0.6507	0.6552	10/10	0.0070
COMT, SULT1E1, ESR1 (c.454 -397T/C)	0.6865	0.6437	7/10	0.0140
SULT1E1, COMT, ESR1 (c.454 -351A/G), ESR1 (c.454 -397T/C)	0.7292	0.6264	10/10	0.0370
**Male**				
COMT	0.5364	0.5947	7/10	0.0170
SULT1E1, COMT	0.5593	0.6069	6/10	0.0050
COMT, SULT1E1, ESR1 (c.454 -397T/C)	0.5896	0.6452	6/10	0.0010
SULT1E1, COMT, ESR1 (c.454 -351A/G), ESR1 (c.454 -397T/C)	0.6267	0.6137	10/10	0.0020

* Interactions were validated based on 1000 permutations.

### Statistics

The demographic and health characteristics of the study subjects were analyzed using Student’s t-test and the Chi-square test. The Hardy-Weinberg equilibrium (HWE) test was assessed by a goodness-of-fit Chi-square test and was performed to examine possible genotyping error for each SNP among controls. Haplotype estimation was restricted to individuals for whom complete genotype data were available across all polymorphic sites, and the highest probability haplotypes estimated using the expectation maximization (EM) algorithm of SAS/Genetics 9.2 (SAS Institute, Cary, NC) were assigned to each study participant. Logistic regression models were used to estimate adjusted odds ratios (ORs) and 95% confidence intervals (CIs) for determining putative high-risk genotypes of each SNP for ischemic stroke. Age, gender, education level, disease history of hypertension, diabetes mellitus, and dyslipidemia, obesity, and cigarette smoking status were adjusted in the models as potential confounders. In addition to traditional multiple logistic regression model to explore high-order gene-gene interactions in susceptibility of young ischemic stroke, we also used several statistical approaches, including the multifactor dimensionality reduction (MDR) software (version 2.0 beta) and MDR-permutation testing (MDRPT) software (version 1.0 beta) and classification and regression tree (CART). The MDR method selects important combinations of variables on the basis of entropy measures for evaluating the information gain (IG) associated with attribute interactions [36]. The patterns of entropy recapitulate the main and/or interaction effect for each model. The CART analysis creates a decision tree that depicts how well each genotype variables predicts patient-control status [37]. Statistical analyses were conducted using SAS package, version 9.2 (SAS Institute, Cary, NC) and R software (version 2.15.0). All statistical tests were based on a two-sided probability.

**Figure 2:**
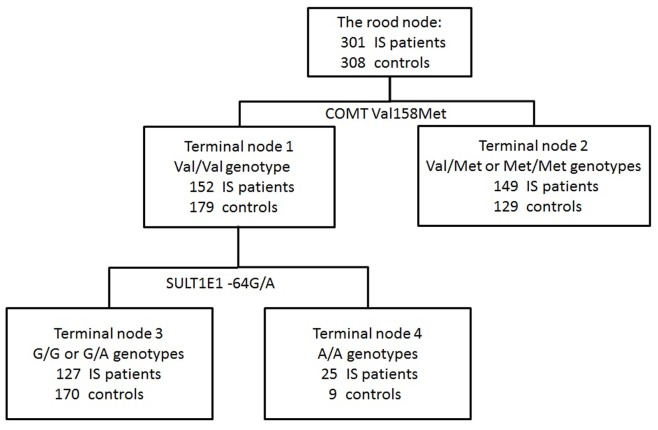
Classification and regression tree analysis of polymorphisms of estrogen metabolic and signaling pathway genes.

## Results

The basic characteristics of the study subjects are illustrated in Table 1. The average age and the distribution of gender were similar between ischemic stroke patients and healthy controls. Cases had higher prevalence of hypertension, diabetes mellitus, cigarette smoking and alcohol drinking behaviors than controls. Mean BMI, WHR, fasting glucose levels, LDLC, and TG were significantly higher in cases than in controls while HDLC were lower in cases compared to controls. To determine the ischemic stroke risk contribution of SULT1E1, COMT and ESR1, we examined whether the genotypic and allelic distribution of the gene differed between the 305 cases and 309 controls (301 cases and 308 controls had results of complete 4 SNPs). The frequencies of all 4 SNPs in the controls agreed with those expected under Hardy-Weinberg equilibrium, suggesting that genotyping error was relatively unlikely. The genotype and allelic analysis for the SNPs yielded no significant differences. However, the permutation test and test for trend showed that the polymorphism of COMT Val158Met was significantly associated with young ischemic stroke susceptibility among females. The results are showed in Table 2. We also found that female patients who carried COMT Met alleles had a significantly higher risk of large artery atherosclerosis (data not shown).

**Table 4:** Risk estimate of classification and regression tree terminal nodes.

Combination of risk genes				Case/Control	OR (95% CI)	P-value	P for interaction
COMT	Val/Val	SULT1E1	GG+GA	127/170	1.0		**0.0171**
			AA	25/9	**6.57(2.55–16.94)**	**<0.0001**	
	Val/Met+Met/Met		GG+GA	138/121	1.41(0.92–2.16)	0.1172	
			AA	11/8	1.51(0.46–4.99)	0.4990	
**Female**							
COMT	Val/Val	SULT1E1	GG+GA	29/54	1.0		**0.0473**
			AA	9/2	**8.9(1.26–63.28)**	**0.0286**	
	Val/Met+Met/Met		GG+GA	45/26	**2.78(1.11–6.97)**	**0.0296**	
			AA	4/5	2.37(0.37–15.35)	0.3647	
**Male**							
COMT	Val/Val	SULT1E1	GG+GA	98/116	1.0		0.1845
			AA	16/7	**5.17(1.73–15.46)**	**0.0033**	
	Val/Met+Met/Met		GG+GA	93/95	1.11(0.68–1.81)	0.6855	
			AA	7/3	1.58(0.26–9.37)	0.6192	

OR was adjustment for age, gender, education level, hypertension, diabetes mellitus, dyslipidemia, obesity, and cigarette smoking.

MDR was used to analyze gene-gene interaction models in young ischemic stroke subjects. The two- to four-way gene-gene interaction models are listed in Table 3. The COMT Val158Met and SULT1E1 -64G/A exhibited the highest testing-balanced accuracy and high cross-validation consistency, especially for females. Figure 1 depicts the interaction maps of all genes based on entropy measures among individual variables for female. The strong interaction effect was found among SULT1E1 -64G/A and COMT Val158Met, which had the IG values of 2.05%. The maps for all subjects and males were shown in Figure S1.

**Table 5:** Odds ratio and 95% confidence interval for the risk of ischemic stroke associated with individual SULT1E1 and COMT and estradiol level.

Genes	Estradiol level*		
SULT1E1/COMT	High	Low	P for interaction
Without risk genotypes	1.0	1.49(0.53–4.23)	**0.0174**
With either one risk genotypes	0.97(0.08–11.43)	**6.13(1.43–26.31)**	

OR was adjustment for age, gender, education level, hypertension, diabetes mellitus, dyslipidemia, obesity, and cigarette smoking.

* High estradiol level defined as median log estradiol level was 1.68 and 1.38 for female and male among healthy controls, respectively.

In the CART analysis, the initial split of the root node was COMT Val158Met, indicating that COMT was the strongest risk factor for young ischemic stroke among all the SNPs. Further inspection of the classification tree structure suggested distinct interaction patterns for subjects with Val/Val genotype and Val/Met and Met/Met genotypes. Among participants with Val/Val genotype, SULT1E1 -64G/A is the strongest risk factor, and the combination of COMT Val/Val genotype and SULT1E1 A/A genotype exhibited the highest risk of ischemic stroke with 73.5% patients rate (OR, 6.57; 95%CI, 2.55–16.94; p<0.0001) (Figure 2 and Table 4). In addition, the logistic regression also showed that there was a two-way interaction in Table 4 (for all subjects, p = 0.0171; for female, p = 0.0473). We further analyzed the interaction effect between serum estradiol level and COMT and SULT1E1 genes on young ischemic stroke patients in Table 5. Relative to the reference group that included subjects with high estradiol level and carried COMT Val/Val genotypes and SULT1E1 G alleles, those whose estradiol level was low and carried either COMT Met allele or SULT1E1 A/A genotype had 6.13-fold risk of ischemic stroke, showing a significant joint effect on risk of young ischemic stroke (P for interaction = 0.0174).

## Discussion

To the best of our knowledge, this study is the first to examine the association between estrogen metabolism and signaling pathway genes, SULT1E1, COMT, and ESR1, and ischemic stroke of the young. The estrogen metabolism genetic polymorphism, COMT Val158Met, was significantly associated with risk of young ischemic stroke among females. Furthermore, we used multianalytic strategies to systemically examine the interaction among these genes. Using different analytic strategy, however, MDR and CART method showed consistent result that there was a strong gene-gene interaction between SULT1E1 -64G/A and COMT Val158Met on the risk of young ischemic stroke. Traditional multiple logistic regression results also showed that there was a significant interaction effect between SULT1E1 -64G/A and COMT Val158Met for development of young ischemic stroke.

Although SULT1E1, a gene encoding an estrogen-metabolizing enzyme, may contribute to individual differences in the biotransformation of this steroid hormone, the relationship between SULT1E1 -64G/A with ischemic stroke was not observed in our study. Owing to low allele frequencies of the three nonsynonymous SNPs among 23 polymorphisms of SULT1E1 identified by Adjei et al. [19], we selected -64G/A located in the promoter region which might influence estrogen sulfotransferase enzyme as the candidate SNPs in our study. We also found that subjects with SULT1E1 -64 G/A AA genotype had significantly lower serum estradiol level than G carriers among healthy controls in our study, especially for females (Table S1). However, the controversial results concerning the association between SULT1E1 -64G/A polymorphism and cancers might be due to the uncertain function of this variant, which might be the reason for non-significant results found in this study [1], [20], [25].

COMT is an important enzyme in the degradation of both catecholamine and estrogens. A non-synonymous G to A base change, COMT Val158Met polymorphism, resulted in the reduction of COMT activity which may impair vascular health in several ways [38], [39]. Several clinical diseases such as preeclampsia [40], hypertension [41], [42] and heart disease [42], [43] have been reported to be associated with this SNP. In addition, growing evidence supports that 2-methoxyestradiol (2-ME), a natural estrogen metabolite produced by COMT, has a potent antiproliferative and antiangiogenic capacity [38], [39] and has direct involvement in redox-regulated signaling as a pro-oxidant [44], thus it could be a possible disease mechanism in the protection against atherosclerosis development. Therefore, these abundant studies support our findings that subjects with COMT Met allele had a significant higher risk of young ischemic stroke among females after 1000 permutation tests.

Estrogen influence multiple organ systems including cardiovascular, reproductive and skeletal systems by binding to specific estrogen receptors located within the nuclei of target cells [45]. Numerous epidemiological and experimental studies indicate the protective roles of estrogens in many forms of cardiovascular and cerebrovascular diseases [46], [47]. However, most studies have focused on the association between ESR1 variants and cardiovascular disease with conflicting results [27]–[30], [48]–[52], and the reason might owe to various study designs. Although our findings including genotype and haplotype analysis (Table S2 and Table S3) reveal no statistically significant risk for ischemic stroke, a gene-environment interaction effect between ESR1 C-A haplotype and serum estradiol level on young ischemic stroke patients was observed (P for interaction = 0.0348, Table S4). The possible mechanism might be that the transcription factor, ERα, interacts directly with specific promoter sequences comprising 15-bp inverted palindromes known as estrogen response elements (EREs) located in the regulatory region of target genes via binding of 17α-estradiol to their classical receptor ERα 53.

In the present study, the power and the possibility of false positives must be considered. According to a relevant range of minor allele frequencies (22–38%), a *post hoc* power calculation can reach to near 80% power to detect an effect size (OR) difference of 1.6 using Power and Sample Size Program (version 3.0.43) 54. In addition, multiple testing is a major concern of this study. Genotype and allelic analysis for each of the 4 SNPs yielded no significant association with ischemic stroke of the young after the Bonferroni correction. The excessively conservative correction of the Bonferroni method might result in the decreased power; therefore, based on 10,000 random permutations, the association between risk of young ischemic stroke and COMT Val158Met among females remained significant.

A major strength of our study is that gene-gene interactions were consistently identified by both MDR and CART analysis. The results were also confirmed by logistic regression approach when controlling for confounding variables simultaneously. To improve the statistical power, the MDR method’s conversion from multiple to single variable resulted in efficient identification of potential gene-gene interactions in relatively small samples 55. In addition, the MDR also reduces the chances of making type I errors as a result of multiple testing through cross validation and permutation testing procedure. The CART analysis is a nonparametric strategy, a decision tree-based data mining to identify specific combinations of genetic factors relating to disease, which requires no assumption of a genetic model. Recent researches have suggested that utilizing multiple complementary analytic approaches can increase statistical power to identify possible gene-gene interactions effectively 56.

There were still some limitations in this study. First, the sample size is relatively small due to difficulty in enrollment of young ischemic stroke patients. Thus, further studies in larger populations are required to validate the findings. Second, we used a candidate approach to select SNPs focusing on the functional variants due to limited budget. However, with more advanced genome-wide association studies exploiting the genetic association study, we may have missed some signals that were not genotyped in the current study. Nevertheless, we cannot rule out the causal markers in the genes we studied. Finally, the menstrual status was acquired for some subjects when the estradiol level was measured. Therefore the misclassification may have occurred when we included females who were in the ovulation stage in the high estradiol level group as the reference. However, this misclassification is non-differential that might dilute the odds ratio and lead to the result toward the null.

In conclusion, these data indicate that COMT Val158Met polymorphism is significantly associated with ischemic stroke risk among females and suggest that gene-gene interaction effect of SULT1E1 -64G/A and COMT Val158Met polymorphisms play more important roles than the individual factors for the development of young ischemic stroke. Moreover, lower estradiol level could increase risk of young ischemic stroke for those who carried either COMT or SULT1E1 risk genotypes.

## Supporting Information

Figure S1 **Interaction map for young ischemic stroke risk.** Values in nodes represent information gain (IG) of individual attribute (main effect). Values between nodes are IGs of each pairwise combination of attributes (interaction effects). A positive entropy (plotted in red or orange) indicates interaction while a negative (plotted in green) indicates redundancy. (a) All subjects. (b) Males. (DOCX)

Table S1 **Association between serum estradiol levels and estrogen metabolism and signaling pathway genes among healthy controls.**
(DOCX)

Table S2 **Estimated haplotype frequencies of ESR1 gene in ischemic stroke patients and healthy controls.**
(DOCX)

Table S3 **Odds ratios between ESR1 haplotype and the risk of ischemic stroke.**
(DOCX)

Table S4 **Odds ratio and 95% confidence interval for the risk of ischemic stroke associated with individual ESR1 C-A haplotype and estradiol level.**
(DOCX)
