# Supplementary material for: Epistasis Analysis for Estrogen Metabolic and Signaling Pathway Genes on Young Ischemic Stroke Patients
Source: PLoS One. 2012 Oct 24;7(10):e47773. doi: 10.1371/journal.pone.0047773 (PMC3480403; doi:10.1371/journal.pone.0047773)
Supplement: Figure S1 — (DOCX) [file pone.0047773.s001.docx]

**Supporting Information**

(a)


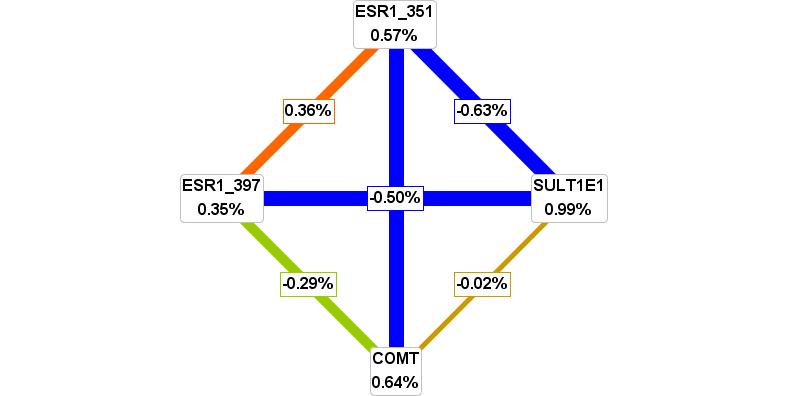


(b)

**
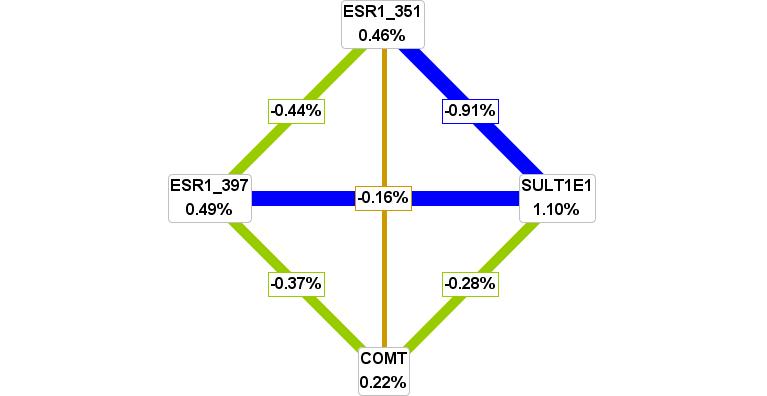
**

Figure S1 Interaction map for young ischemic stroke risk. Values in nodes represent information gain (IG) of individual attribute (main effect). Values between nodes are IGs of each pairwise combination of attributes (interaction effects). A positive entropy (plotted in red or orange) indicates interaction while a negative (plotted in green) indicates redundancy. (a) All subjects. (b) Males.
