# Supplementary material for: Epistasis Analysis for Estrogen Metabolic and Signaling Pathway Genes on Young Ischemic Stroke Patients
Source: PLoS One. 2012 Oct 24;7(10):e47773. doi: 10.1371/journal.pone.0047773 (PMC3480403; doi:10.1371/journal.pone.0047773)
Supplement: Table S1 — (DOCX) [file pone.0047773.s002.docx]

**Supporting Information**

Table S1 Association between serum estradiol levels and estrogen metabolism and signaling pathway genes among healthy controls

|  |  | Log transformed estradiol level | | | | | | | |
| --- | --- | --- | --- | --- | --- | --- | --- | --- | --- |
| Gene | Genotypes | Total | |  | Female | |  | Male | |
|  |  | Mean (SD) | P-value |  | Mean (SD) | P-value |  | Mean (SD) | P-value |
| SULT1E1 | GG+GA | 1.41(0.40) | **0.0022** |  | 1.48 (0.57) | **0.0070** |  | 1.37 (0.23) | 0.2131 |
| -64G/A | AA | 1.19(0.32) |  |  | 1.08 (0.38) |  |  | 1.30 (0.21) |  |
|  |  |  |  |  |  |  |  |  |  |
| COMT1 | Val/Val+Val/Met | 1.38 (0.40) | 0.7485 |  | 1.42 (0.57) | 0.6307 |  | 1.36 (0.23) | 0.8941 |
| Val158Met | Met/Met | 1.41 (0.37) |  |  | 1.54 (0.58) |  |  | 1.37 (0.29) |  |
|  |  |  |  |  |  |  |  |  |  |
| ESR1 | TT+TC | 1.39 (0.40) | 0.6626 |  | 1.43 (0.57) | 0.5181 |  | 1.36 (0.24) | 0.7561 |
| c.454 -397T/C | CC | 1.36 (0.37) |  |  | 1.34 (0.56) |  |  | 1.38 (0.19) |  |
|  |  |  |  |  |  |  |  |  |  |
| ESR1 | AA+AG | 1.39 (0.40) | 0.8447 |  | 1.43 (0.57) | 0.2849 |  | 1.36 (0.23) | 0.1714 |
| c.454 -351A/G | GG | 1.37 (0.31) |  |  | 1.15 (0.36) |  |  | 1.44 (0.27) |  |
|  |  |  |  |  |  |  |  |  |  |
| ESR1 C-A haplotype | 0 copies | 1.41 (0.39) | 0.1572 |  | 1.45 (0.57) | 0.4432 |  | 1.38 (0.22) | 0.0953 |
|  | >=1 copies | 1.34 (0.41) |  |  | 1.37 (0.56) |  |  | 1.32 (0.26) |  |
