# Supplementary material for: Epistasis Analysis for Estrogen Metabolic and Signaling Pathway Genes on Young Ischemic Stroke Patients
Source: PLoS One. 2012 Oct 24;7(10):e47773. doi: 10.1371/journal.pone.0047773 (PMC3480403; doi:10.1371/journal.pone.0047773)
Supplement: Table S2 — (DOCX) [file pone.0047773.s003.docx]

**Supporting Information**

Table S2 Estimated haplotype frequencies of ESR1 gene in ischemic stroke patients and healthy controls

| Haplotypes | Cases (%) | Controls (%) | OR (95% CI) | OR^a^ (95% CI) |
| --- | --- | --- | --- | --- |
| TA | 57.8 | 61.0 | 1.0 | 1.0 |
| CG | 22.1 | 21.0 | 1.11(0.89-1.37) | 1.13(0.86-1.49) |
| CA | 17.7 | 16.6 | 1.13(0.89-1.42) | 1.13(0.83-1.52) |
| TG | 2.39 | 1.40 | --- | --- |

a: adjustment for age, gender, education level, hypertension, diabetes mellitus, dyslipidemia, obesity, and cigarette smoking
