# Supplementary material for: Epistasis Analysis for Estrogen Metabolic and Signaling Pathway Genes on Young Ischemic Stroke Patients
Source: PLoS One. 2012 Oct 24;7(10):e47773. doi: 10.1371/journal.pone.0047773 (PMC3480403; doi:10.1371/journal.pone.0047773)
Supplement: Table S3 — (DOCX) [file pone.0047773.s004.docx]

**Supporting Information**

Table S3 Odds ratios between ESR1 haplotype and the risk of ischemic stroke

|  |  |  | Prevalence in controls, % | 0 copies | | 1 copy | | | | 2 copies | | | |  | Additive model |  |
| --- | --- | --- | --- | --- | --- | --- | --- | --- | --- | --- | --- | --- | --- | --- | --- | --- |
| Haplotype |  |  |  | Case/Control | OR | Case/Control | OR | 95% CI | P-value | Case/Control | OR | 95% CI | P-value | OR | 95% CI | P-value |
| ESR1 | H1 | TA | 86.1 | 50/49 | 1.0 | 150/157 | 0.70 | 0.39-1.26 | 0.2332 | 101/109 | 0.57 | 0.31-1.06 | 0.0749 | 0.77 | 0.57-1.03 | 0.0798 |
|  | H2 | CG | 37.2 | 189/194 | 1.0 | 105/98 | 1.16 | 0.76-1.77 | 0.5038 | 7/17 | 0.85 | 0.29-2.51 | 0.7737 | 1.06 | 0.75-1.51 | 0.7340 |
|  | H3 | CA | 29.8 | 203/217 | 1.0 | 83/85 | 1.18 | 0.74-1.86 | 0.4899 | 15/7 | 2.81 | 0.91-8.69 | 0.0736 | 1.35 | 0.93-1.96 | 0.1119 |
| **Female** |  |  |  |  |  |  |  |  |  |  |  |  |  |  |  |  |
| ESR1 | H1 | TA | 87.5 | 14/11 | 1.0 | 49/46 | 0.85 | 0.27-2.73 | 0.7877 | 24/31 | 0.32 | 0.09-1.18 | 0.0868 | 0.54 | 0.29-1.02 | 0.0582 |
|  | H2 | CG | 34.1 | 52/58 | 1.0 | 34/26 | 2.01 | 0.83-4.87 | 0.1227 | 1/4 | 0.34 | 0.02-5.95 | 0.4603 | 1.34 | 0.65-2.76 | 0.4253 |
|  | H3 | CA | 32.96 | 58/59 | 1.0 | 25/26 | 1.66 | 0.64-4.30 | 0.2956 | 4/3 | 1.96 | 0.26-14.87 | 0.5167 | 1.53 | 0.74-3.19 | 0.2519 |
| **Male** |  |  |  |  |  |  |  |  |  |  |  |  |  |  |  |  |
| ESR1 | H1 | TA | 85.5 | 36/32 | 1.0 | 101/111 | 0.70 | 0.35-1.40 | 0.3131 | 77/78 | 0.67 | 0.33-1.39 | 0.2829 | 0.85 | 0.60-1.20 | 0.3556 |
|  | H2 | CG | 38.5 | 137/136 | 1.0 | 71/72 | 1.02 | 0.62-1.68 | 0.9337 | 6/13 | 1.01 | 0.30-3.40 | 0.9861 | 1.02 | 0.67-1.53 | 0.9427 |
|  | H3 | CA | 28.5 | 145/158 | 1.0 | 58/59 | 1.07 | 0.63-1.85 | 0.7950 | 11/4 | 3.57 | 0.82-15.59 | 0.0906 | 1.32 | 0.84-2.06 | 0.2281 |

OR was adjusted for age, gender, education level, hypertension, diabetes mellitus, dyslipidemia, obesity, and cigarette smoking
