# Supplementary material for: Epistasis Analysis for Estrogen Metabolic and Signaling Pathway Genes on Young Ischemic Stroke Patients
Source: PLoS One. 2012 Oct 24;7(10):e47773. doi: 10.1371/journal.pone.0047773 (PMC3480403; doi:10.1371/journal.pone.0047773)
Supplement: Table S4 — (DOCX) [file pone.0047773.s005.docx]

**Supporting Information**

Table S4 Odds ratio and 95% confidence interval for the risk of ischemic stroke associated with individual ESR1 C-A haplotype and estradiol level

| Genes |  | Estradiol level | |  |
| --- | --- | --- | --- | --- |
| ESR1 C-A haplotype |  | High | Low | P for interaction |
| 0 copies |  | 1.0 | **3.49(1.17-10.40)*** | **0.0348** |
| ≥ 1 copy |  | 1.87(0.46-7.65) | 2.96(0.64-13.68) |  |

OR was adjusted for age, gender, education level, hypertension, diabetes mellitus, dyslipidemia, obesity, and cigarette smoking

High estradiol level defined as median log estradiol level was 1.68 and 1.38 for female and male among healthy controls, respectively.
